# Review of "Basic Orthopaedic Biomechanics and Mechano-Biology" 3rd Edition, by Van C. Mow and Rik Huiskes

**DOI:** 10.1186/1475-925X-4-28

**Published:** 2005-04-20

**Authors:** Eduardo Abreu

**Affiliations:** 1 Children's Hospital of Boston, Department of Orthopaedic Surgery, 300 Longwood Street, Boston MA 02115, USA

This is the third edition of the already well-known book "Basic Orthopaedic Biomechanics." In addition to restructured chapters, new material reflecting current trends in the field has been included in this new edition, and "Mechano-Biology" was added to the title. These most appropriate revisions help to make this a book that is at the same time both "classic" and "up-to-date."

This book begins with a brief history of science and orthopaedic biomechanics, where the authors discuss how the conflict and inconsistencies of accepted paradigms are challenged by new tools and experiments, leading to new ways of addressing problems in the field. Although there are often "titanic" struggles involved in making such paradigm shifts, conflict is often a necessary first step in the advancement of knowledge. This book not only presents the current knowledge in the field, but also has the objective of motivating new questions that could result in future advances.

An exceptional team of contributing authors collaborated in writing the various chapters covering different topics in orthopaedic biomechanics and mechano-biology including: analysis of muscle and joint loads, kinematics, biomechanics of musculoskeletal tissues (bone, cartilage, meniscus, tendons and ligaments), cartilage and bone tissue engineering, spine and artificial joint biomechanics, etc. There is also an excellent chapter on biomaterials. The chapters are well-written and the figures are informative and of good quality. Each chapter is complemented by an extensive list of references.

An important characteristic of this book is that it can be used by any student or researcher in orthopaedic biomechanics, regardless of instructional level or background. Basic concepts are always well explained before they are used. For example, in chapter 1 Newton's laws are reviewed before the forces involved in the analysis of muscle and joint systems are calculated. Being an interdisciplinary subject, many times topics in biomechanics make use of concepts from biology and biochemistry. Whenever such cross-disciplinary ideas present they are well explained; for example, proteoglycans in chapter 5 and intracellular signaling pathways in chapter 6.

In summary, this is an excellent book in orthopaedic biomechanics that will greatly benefit all members of the biomechanics community. It can be used as a text for beginning and advanced students, as well as a reference for both students and researchers at all levels, or for those who just want to learn something about biomechanics.

